# Strategies to Improve Therapeutic Adherence in Polymedicated Patients over 65 Years: A Systematic Review and Meta-Analysis

**DOI:** 10.3390/pharmacy12010035

**Published:** 2024-02-17

**Authors:** Natalia Burgos-Alonso, María Torrecilla, Aitziber Mendiguren, Marta Pérez-Gómez Moreta, Cristina Bruzos-Cidón

**Affiliations:** 1 Public Health Department, Faculty of Medicine and Nursing, University of the Basque Country UPV/EHU, 48940 Leioa, Spain; 2 Pharmacology Department, Faculty of Medicine and Nursing, University of the Basque Country UPV/EHU, 48940 Leioa, Spain; 3 Nursing I Department, Faculty of Medicine and Nursing, University of the Basque Country UPV/EHU, 48940 Leioa, Spain

**Keywords:** elderly, polymedication, adherence, meta-analysis, systematic review

## Abstract

Background: Part of the population over 65 years of age suffer from several pathologies and are therefore polymedicated. In this systematic review and metanalysis, we aimed to determine the efficacy of several strategies developed to improve adherence to pharmacological treatment in polymedicated elderly people. Design: Web Of Science, PubMed and the Cochrane Library were searched until 2 January 2024. In total, 17 of the 1508 articles found evaluated the efficacy of interventions to improve adherence to medication in polymedicated elderly patients. Methodological quality and the risk of bias were rated using the Cochrane risk of bias tool. Open Meta Analyst^®^ software was used to create forest plots of the meta-analysis. Results: In 11 of the 17 studies, an improvement in adherence was observed through the use of different measurement tools and sometimes in combination. The most frequently used strategy was using instructions and counselling, always in combination, in a single strategy used to improve adherence; one involved the use of medication packs and the other patient follow-up. In both cases, the results in improving adherence were positive. Five studies using follow-up interventions via visits and phone calls showed improved adherence on the Morisky Green scale compared to those where usual care was received [OR = 1.900; 95% CI = 1.104–3.270] (*p* = 0.021). Discussion: There is a high degree of heterogeneity in the studies analyzed, both in the interventions used and in the measurement tools for improving adherence to treatment. Therefore, we cannot make conclusions about the most efficacious strategy to improve medication adherence in polymedicated elderly patients until more evidence of single-intervention strategies is available.

## 1. Introduction

It is estimated that 20% of the resident population in Europe in 2020 are 65 years old or older [1]. This aging of the population would result in changes and adaptations in society to improve the quality of life and welfare of this population category. The population over 65 years is characterized by suffering from more than one concomitant disease [2,3], generally chronic, such as hypercholesterolemia, hypertension, diabetes, heart failure, renal failure, arthritis or those affecting the central nervous system such as Alzheimer’s and Parkinson’s disease or dementia among others [4,5]. Pluripathology leads to a lesser degree of functionality of the elderly population, which influences the difficulty in carrying out the activities of their daily lives [6]. Patients are chronically prescribed to treat their different pathologies and improve their health-related quality of life [7,8,9]. Studies show that a high percentage of patients with chronic treatment are non-adherent to treatment [10]. 

Non-adherence is a public health problem, described as the failure to follow the instructions offered by the healthcare professional regarding the use of medication and lifestyle habits aimed at treating a pathology. An increase in the number of medications is considered a risk factor for non-adherence. Indeed, in polymedicated people, multiple problems related to medication utilization can appear, such as difficulty in the recognition of medication packaging, dosage error, mistakes in the frequency of dosing, misunderstanding prescription changes or difficulty in correct handling of the packaging, which are considered as a lack of adherence [11]; all of these aspects are included in the lack of adherence [12]. Non-adherence leads to weaker control of the disease and, consequently, a poorer quality of life for the elderly patient [13,14]. It also means an increase in health care costs, since patients who do not receive correct medication control are more likely to suffer from adverse effects and consequently make more visits to the health center or require hospital admission [15,16,17]. 

Along these lines, different strategies have been developed with the aim of improving adherence to treatment [18,19,20] which involve healthcare professionals, including medical, nursing and pharmacy staff. These strategies include the prescription of prolonged-release drugs to reduce the frequency of doses, patient education on their pathology and the need for treatment and its dosage [21]. In addition, calendars, electronic or telephone reminders [22] and personalized dosing systems (PDS) can also be used [23]. 

To date, most studies have focused on studying adherence to a single pharmacological group so that this population group is not covered and the applicability of adherence improvement tools to the elderly who received chronic treatments based on three or more drugs has not been frequently analyzed [18,19,24,25,26]. In fact, although the study performed by Cross et al. [27] aimed to assess adherence in patients over 65 years old, in many of the studies included in their review the patients were younger than 65 years old.

In addition, the present study also allows us to include some recent studies that update and expand our knowledge on the best strategies for adherence to treatment in this population. Therefore, the aim of this systematic review was to study the impact of the different interventions carried out to improve adherence to treatment in the polymedicated elderly population (over 65 years old) until January 2024.

## 2. Materials and Methods

This systematic review was performed according to the preferred reporting items for systematic review and meta-analysis (PRISMA) guidelines [28]. The methodology used is in line with the methods developed by the Cochrane Collaboration [29] to search, retrieve, appraise the quality and synthesize the findings of randomized controlled trials (RCTs). For the purposes of this review, the term polypharmacy was defined as the use of 3 or more drugs. Registered in PROSPERO as CRD42021267336.

### 2.1. Search Methods

Two authors independently identified eligible studies indexed in the Cochrane Library (CENTRAL), PubMed (Medline) and Web of Science (ISI) database published in English or Spanish up to January 2024. Studies were sought using the combined search terms ‘adherence’, ‘compliance’, ‘intervention’, ‘strategy’, ‘polypharmacy, ‘older’, ‘aged’ and ‘elderly’ (Supplementary Table S1). To further substantiate the searching process, a manual search of relevant journals (Patient and Education Counseling, Atención Primaria, Farmacia Hospitalaria) and references of retrieved papers was undertaken, which was especially important for those journals less likely to be indexed. Studies that matched the key words were included in the review. 

### 2.2. Inclusion and Exclusion Criteria

Inclusion criteria were as follows: population, polymedicated participants over the age of 65 years; intervention, an adherence strategy; studies including a control group; outcomes, data on adherence using, i.e., pill count, self-reported questionnaire, ‘renewal or refill’, “blood level analysis” and “rehospitalisation”. Adherence was reported as a primary or a secondary outcome. Any study that did not report adherence was excluded.

Papers assessing adherence to medication in patients who suffer from a single disease condition were excluded. Protocols, conference, abstracts, academic thesis, editorials, commentaries and opinion articles were excluded. Review articles were excluded, although they were used for cross-checking relevant primary papers.

### 2.3. Data Management

The bibliographic software EndnoteX7.4^®^ was used to store and organize all results from the bibliographic databases. Microsoft Excel 2016 was used to support the selection of papers and data extraction process, and Microsoft Word 2016 was used for the quality appraisal process.

The heterogeneity between studies was assessed using the I2 statistic. Studies with an I2 statistic > 50% were considered to have significant heterogeneity. Pooled analyses were considered statistically significant when the *p* value < 0.05. OpenMeta Analyst software was used to create forest plots and analyze the included studies.

### 2.4. Study Selection

All identified articles were independently screened by two members of the review team. First, the title and abstract of all papers were screened to determine their relevancy, followed by a full-text reading of all remaining papers.

Any disagreement on inclusion was resolved by reaching a consensus through discussion or adding a third reviewer. Reviewers were not blinded to a paper’s author/s.

### 2.5. Data Extraction

An a priori data collection instrument was piloted by the review team. Data extraction was undertaken by one reviewer and double checked for accuracy by a second. Any discrepancies were resolved through discussion and further scrutiny of the included paper. Extracted data were as follows: study design, setting, study duration (months), total number of participants (n = intervention group), type of intervention/person leading, usual care (if applicable), adherence outcome and results.

### 2.6. Risk of Bias in Individual Studies

On one hand, the risk of bias for controlled trials with randomization was assessed using the updated Cochrane risk of bias 2 (RoB 2). On the other hand, the risk of bias in non-randomized studies of interventions (ROBINS-I) was used for non-randomized studies. The sources of bias included in the RoB2 Cochrane library were adequate sequence generation, adequate assignment to intervention, the effect of blinding the intervention, bias due to missing outcome information and bias in the selection of the reported result [29]. The sources of bias included in ROBINS-I were the bias of the classification of interventions inappropriately, bias due to deviations from intended interventions, bias due to missing data, bias in the measurement of outcomes and bias in the selection of the reported results [30]. Two reviewers independently examined the risk of bias, by means of a defined questionnaire which included evaluation of the cited domains, each study was scored as a high, low or unclear risk of bias. Discrepancies between reviewers were solved by a third author. 

### 2.7. Quantitative Analysis

A meta-analysis was performed to estimate the overall effects of medication review on adherence using the odds ratio (OR) with a 95% confidence interval (CI). Because the true effects were expected to be related but not the same for the included studies, a random-effects model was used for all analyses. Statistical heterogeneity between studies was assessed using the χ^2^ test and I2 index. Once statistical heterogeneity was observed, potential sources of heterogeneity were explored accordingly. Meta-analyses were conducted using OpenMeta [Analyst] software [31].

## 3. Results

**Figure 1:**
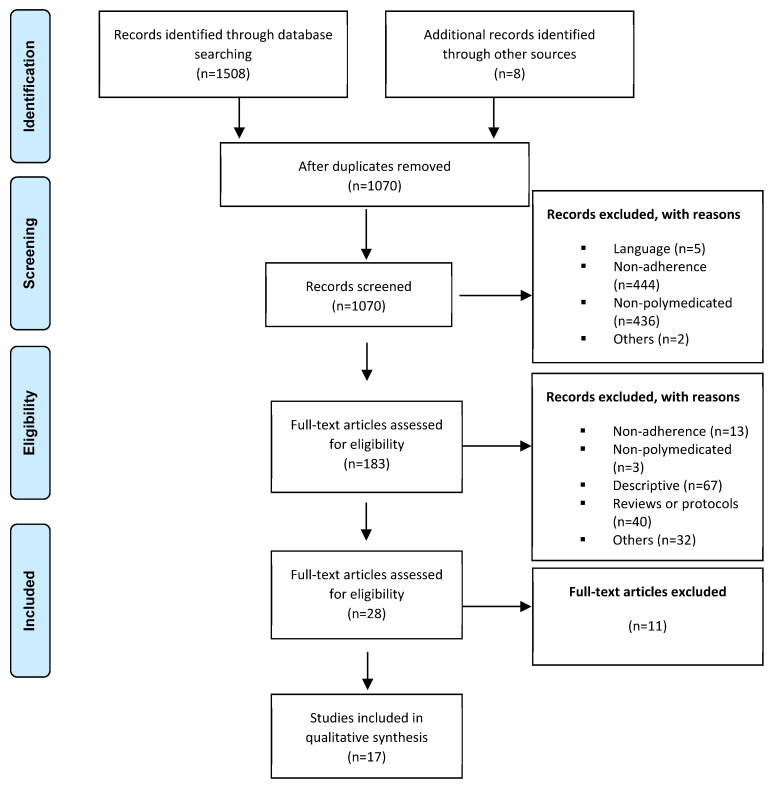
Flow diagram of screening process and reasons for exclusion of studies (PRISMA).

A total of 1508 unique papers were identified from the database searching and 8 more by reverse search. After screening their title and abstracts, 182 were eligible for further review. Of these articles, and after full-text reading, author citation and reference list evaluation, a total of 17 met the eligibility criteria and underwent quality appraisal and data extraction processes [9,21,32,33,34,35,36,37,38,39,40,41,42,43,44,45,46]. The literature review screening process is summarized in Figure 1.

### 3.1. Studies and Participants

Included studies were carried out in: Canada [46], China [9], Germany [40], India [33], Spain [34,37,38,43], Switzerland [36], Malaysia [44], the United Kingdom [32,41,42,45] and the USA [35,39]. One study was conducted across seven countries [21]. Twelve studies [9,21,33,34,35,36,38,39,41,43,44,45] were RCTs and five were quasi-experimental [32,37,40,42,46].

A total of 6463 participants were included in the review [9,21,32,33,34,35,36,37,38,39,40,41,42,43,44,45,46]. Four studies involved 100 or fewer participants [32,33,39,43], six studies involved between 100 and 300 participants [35,37,38,42,44,45] and seven studies included more than 300 participants [9,21,34,36,40,41,46]. In all studies, the intervention was directed to the patients. The mean/median age of included patients in the intervention groups ranged from 67.2 [36] to 84.0 [41] years and the mean/median age of included patients in the control groups ranged from 66.9 [33] to 84.0 [41] years, and 32.7% (2112/6463) of the patients were female. The minimum number of medications taken by polymedicated patients ranged from 3 [38,39,46] to 8 [34], being in most cases 4 or 5 prescribed drugs [9,21,32,35,36,37,40,41,43,44,45]. In 2 studies [33,42], the authors did not specify the minimum number of medicines. The mean of number of medications taken by patients overall was 7.48 (Table 1).

### 3.2. Types of Interventions

Seven studies [32,33,34,35,37,39,40] were conducted in a primary care center, including a geriatric health center, five in community pharmacies [21,36,44,45,46], three studies were conducted in the hospital (including posthospitalization) [9,38,43], one in a hospital and community pharmacy [41] and one of them was conducted in a clinical pharmacy [42].

The healthcare professional leading the intervention varied significantly. Eight studies [9,32,36,39,41,42,43,46] were led by a community or clinical pharmacist, while five studies [21,34,35,44,45] were co-led by a general practitioner (GP) and pharmacists, two studies [37,40] were led by a GP or health care provider, one study [33] was led by a researcher and another study [38] was led by a community nurse.

Interventions varied in duration, ranging from 2 [38,43] to 24 months [9] (Table 1). We summarized the interventions into seven groups, as follows: instructions and counselling in 10 studies [32,33,35,36,37,38,40,41,43,46], an education program in 6 studies [9,21,35,36,44,45], a simplifying regimen or discharge in 4 studies [21,32,34,45], reminder systems in 5 studies [21,32,35,36,43], monitoring in 11 studies (4 studies implemented home visits [32,37,41,45], 4 studies implemented visits [33,34,36,44], 5 studies implemented telephone calls [9,33,36,42,46]), medication review was conducted in 3 studies [40,41,46] and medication packages were used in 3 studies [35,38,39]. Eight of the studies combined two interventions [9,33,34,37,38,40,43,44], four combined three interventions [21,41,45,46] and three combined four interventions [32,35,36]. Only two studies assessed a single intervention [39,42] (Table 2).

### 3.3. Effect of Interventions

Significant improvements were reported in 5 [32,33,36,37,43] out of 10 [32,33,35,36,37,38,40,41,43,46] studies that assessed giving instructions to the patient on medication use as an adherence strategy. 

In two of the nine studies, the use of instructions was combined with educational programs [35,36]. Only one of the two showed improved adherence [36]. Interestingly, all of them combined instructions with other strategies such as medication discharge [32], reminder systems [32,36,43] and monitoring [32,33,37].

In the case of Hanlon et al. [35], a combination of educational tools, reminder systems and dosing systems were used but no significant improvement in patients’ adherence to treatment was observed. Similarly, Morales et al. [38] who also combined education with dosing systems, did not observe an improvement in adherence. Muth et al. [40], Nazareth et al. [41] and Volume et al. [46] added patient education to medication review without significant improvement in adherence. In addition, Nazareth et al. [41] and Volume et al. [46] included patient follow-up, but again no significant change in adherence was obtained (Table 1).

In the studies of Bernsten et al. [21], Shim et al. [44] and Wu et al. [9], educational programs were used with a significant gain in adherence to treatment by patients who had received the intervention. In the Bernsten et al. [21] study, positive results were obtained from the combination of the education program, medication discharge and reminder systems. In contrast, in the Sturgess et al. [45] study, the use of educational programs, medication discharge and monitoring failed to improve patient adherence. In line with this, in the studies of Shim et al. [44] and Wu et al. [9], a significant improvement in adherence was not achieved after using an educational program and monitoring.

Finally, in the Campins et al. [34] study, improved adherence was achieved through medication discharge and monitoring. In the Murray et al. [39] study, which only used a dosing tool, positive results in adherence were obtained. Similarly, Odeth et al. [42] showed improved adherence through patient monitoring (Table 1 and Table 2).

### 3.4. Adherence Measurement

The tool used to measure adherence varied between studies although not to the same extent as the intervention tool. The Morisky Green scale or an adaptation was used in seven studies [21,33,34,37,38,40,44]; medication possession ratio/pill count in six studies [9,32,36,39,40,45]; self-report in five studies [35,36,41,43,46]; health care utilization in two studies [43,45]; and a medication adherence rating scale in one study [42] (Table 1). In the studies, different tools were used or combined to measure adherence to the treatment (Table 3).

### 3.5. Risk of Bias of Selected Studies

The risk of bias of each randomized clinical trial is shown in Table 4. Briefly, the risk of bias regarding the domain “sequence generation” was considered low in nine trials [9,33,34,35,36,37,40,41,43,44], unclear in three [38,39,45] and the remaining studies were categorized as high risk [21,37,46]. The risk of bias arising from the domain “allocation concealment” was considered low in five trials [9,34,36,40,43], high risk as assessed at three studies [21,37,46] and the rest of the studies did not specify this domain [33,35,38,39,41,44,45].

Concerning performance bias, the blinding procedure was not found in any of the studies [9,21,33,34,35,36,38,39,40,41,42,43,44,45,46]. 

The risk of attrition bias was rated as low in ten trials [33,34,35,36,37,38,39,40,44,45], was unclear in two studies [41,43] and high in the remaining studies [9,21,35,46]. Reporting bias was rated as low in twelve trials [9,21,33,34,35,36,37,41,43,44,45,46]. The risk of other bias was rated as low in twelve trials [9,21,33,34,36,37,38,40,41,43,44,45] and unclear in three studies [35,39,46] (Table 4).

Regarding the two non-randomized clinical studies in which the ROBINS-I tool was used, we observed that the study of Al-Rashed et al. [32] showed little or no risk in the confounding bias, selection of participant in the study and classification of intervention bias, while Odeth et al. [42] revealed a risk in the mentioned bias items (Table 5).

### 3.6. Meta-Analysis Results

The studies that were selected for meta-analysis were those that showed homogeneity at the methodological level, both in the intervention and in the outcome, in the effect of visits or telephone call (self-reported questionnaire of adherence: >80% or <120%). 

**Figure 2:**
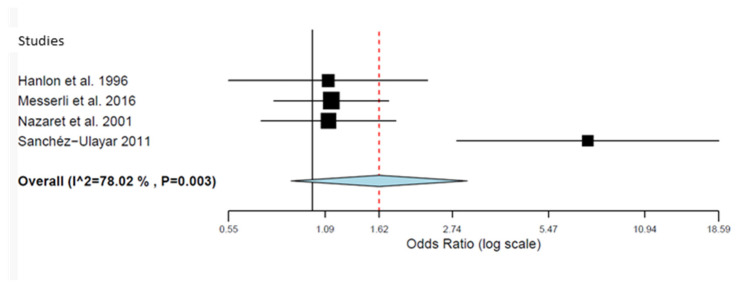
Forest plot of the effect of visits and telephone call interventions (self-reported adherence (80–120%)) [35,36,41,43].

Four studies [35,36,41,43] reported the effects of visits and telephone calls on adherence in 907 elderly patients with polypharmacy (Table 6 and Figure 2). The overall result indicates that there is no difference between the effect obtained in patients who received visits or telephone call and that observed in patients who received usual care (OR = 1.900; 95% CI = 1.104–3.270) (*p* = 0.138), although a substantial heterogeneity was observed (I2 = 78.02, *p* = 0.003). 

### 3.7. Effect of Visits or Telephone Call (Morisky Green Scale)

**Figure 3:**
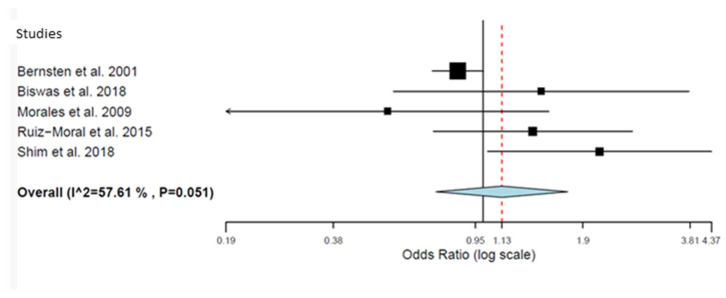
Forest plot of the effect of visits and telephone calls (Morisky Green scale) [21,33,37,38,44].

Five studies [21,33,37,38,44] reported the effects on adherence by the Morisky Green scale on elderly patients with polypharmacy (Table 7 and Figure 3). Overall, the results suggest an improvement in adherence from visits or telephone calls compared to those receiving usual care (OR = 1.616; 95% CI = 0.857–3.048) (*p* = 0.021), although a substantial heterogeneity was observed [*p* < 0.003, I2 = 75.43].

**Table 1:** Characteristics and results of included studies.

Country, Data and Reference of the Study	Design	Setting	Duration (Months)	Number of Participants (n_ Intervention Group)	Age [Mean Years (SD)] (% Male)	Type of Intervention/Person Leading	Outcome Measurement	Results (*p* Value)
Al-Rashed et al., 2002 England [32]	Quasi-experimental study	Primary care centers	3	83 (n = 43)	EG = 80.2 (5.7) CG = 81.1 (5.8) EG = 36.4 CG = 39	Counselling, pre-discharged, medication reminder system (reminder cards)/2 home visits/clinical and researcher pharmacist.	Adherence tablet count	Compliance EG = 40.4% to 70.0% and CG = 15.9% to 15.8%; *p* < 0.001.
Bernsten et al., 2001 7 European countries [21]	RCT	Community pharmacy	18	2454 (n = 1290)	EG = 74 (8) CG = 74 (8) EG = 42.1 CG = 42.	Educational program, simplifying regime, medication reminder system (as compliance strategy)/community pharmacist and general practitioner.	Based on Morisky Green (4 items)	Changed from being noncompliant to compliant EG = 15.2% and CG = 12.2%; *p* = 0.028.
Biswas et al., 2018 India [33]	RCT	Semi urban community	18	75 (n = 40)	EG = 68.4 (5.77) CG = 66.89 (6.77) EG = 77.5 CG = 5.3	Counselling, visits, time table charting and reinforcement, direct or telephonic follow-up/investigator.	MMAS-8 Subjects without missing any dose	Changes in MMAS-8 score EG = 2.45 to 0.39 and CG = 2.97 to 1.03; *p* < 0.001. Subjects without missing any dose: 40% to 51% and 31% to 32%; *p* = 0.023.
Campins et al., 2017 Spain [34]	RCT	Primary care centers	12	503 (n = 252)	EG = 79.16 (5.50) CG = 78.78 (5.46) EG = 39.7 CG = 42.6	Simplifying regime (STOP-START criteria) visits (supervision)/clinical pharmacist and general practitioner.	Morisky Green	Medication adherence: EG = 76.4% and CG = 64.1%; *p* = 0.005.
Hanlon et al., 1996 USA [35]	RCT	Primary care centers	12	208 (n = 105)	EG = 69.7 (3.5) CG = 69.9 (4.1) EG = 98.1 CG = 100	Instructions, education written material, medication reminder system, packages or calendars when necessary/clinical pharmacist and general practitioner.	Patient self-report	Compliance EG = 73% to 77.4% and CG = 74% to 76.1%; *p* = n.s.
Messerli et al., 2016 Switzerland [36]	RCT	Community pharmacy	7	450 (n = 218)	EG = 67.2 (11.52) CG = 67.1 (11.56) EG = 45.9 CG = 46.1	Counselling, education, medication reminder system, visits, telephone interviews/community pharmacist.	Number of medication missed/Number of medication could be missed. Subjective adherence: self-reported questionnaire	Number of patients reporting an improved subjective adherence between T-0 and T-2 was significantly higher in the EG (nImprovement = 30; nWorsening = 14) than in the CG (nImprovement = 20; nWorsening = 24; *p* = 0.028). Insufficient adherence to at least one medicine (n = 69, 26.7%).
Moral et al., 2015 Spain [37]	Cluster RCT	Primary care centers	6	150 (n = 70)	EG = 75.6 (5.9) CG = 76.1 (5.8) EG = 30 CG = 32.1	Counselling (motivation) and visits at home/health care providers.	Adherence tablet count (>80% or <110%)	The proportion of subjects changing to adherence was 7.6% higher in the EG; *p* < 0.001.
Morales et al., 2009 Spain [38]	RCT	Hospital	2	182 (n = 89)	EG = 77.08 (93–61) GC = 77.39 (70–20) EG = 28.1 CG = 31.2	Counselling and medication package (pillbox with instructions)/nurse.	Morisky Green	Medication adherence improved EG = 6.74% to 13.5% and GC = 11.8% to 14%; *p* = 0.18.
Murray et al., 1993 USA [39]	RCT	Geriatric health center	6	32(EG1 = 10; EG2 = 9)	EG1 = 72.9 (6.1) EG2 = 72.5 (10.1) CG = 71.3 (5.5) EG1 = 19 EG2 = 20 CG = 25	Medication package (unit-of-use package)/pharmacist.	Adherence tablet count	Compliance EG1 = 82.6%, EG2 = 92.6% and GC = 79%; *p* = 0.017.
Muth et al., 2018 Germany [40]	Cluster RCT	Primary care centers	9	505 (n = 252)	EG = 51.9 (7.0) CG = 50.2 (7.6) EG = 58 CG = 56	Instructions and medication review,/general practitioner and healthcare assistant.	Observed adherence: dose score	Number and percentage of deviating patients EG = 63.3% and CG = 54.5%; *p* = n.s.
Nazareth et al., 2001 United Kingdom [41]	RCT	Hospital and community pharmacy	6	362 (n = 181)	EG = 84 (5.2) CG = 84 (5.4) EG = 38 CG = 34	Counselling, home visit, medication review, medication review/clinical and community pharmacist.	Prescribed medicine interview. Validated self-report semi-structured interview. ** mean (SD)	EG = 0.8(0.31) to 0.78(0.3) and CG = 0.77(0.3) to 0.78(0.3); *p* = n.s.
Odeh et al., 2019 United Kingdom [42]	Quasi-experimental study	Clinical pharmacy	3	211 (n = 131)	EG = 68.8 (12.4) CG = 68.8 (12.4) EG = 52 CG = 52	Telephone follow-up/clinical pharmacist	MARS	EG = Mean difference was 1.4 (pre: 22.7 vs. post 24.1; *p* < 0.001). In total, 28% of patients reported the same score before and after the intervention.
Sanchez-Ulayar et al., 2011 Spain [43]	RCT	Hospital	2	100 (n = 50)	EG = 75 (11) CG = 11 (10) EG = 42 CG = 42	Medication reminder system/clinical pharmacist	Adherence tablet count (self-reported)	Became totally compliant EG = 70.7% and CG 19.5%; *p* < 0.001.
Shim et al., 2018 Malaysia [44]	RCT	Community pharmacy	6	152 (n = 73)	EG = 72.0 (7.0) CG = 171.0 (6.0) * EG = 42 CG = 45	Education and visits/pharmacist and general practitioner	MALMAS	Non-adherence (<6 score): EG 35.6% to 66.9% and GC = 32.9% to 31.6%; *p*< 0.001.
Sturgess et al., 2003 United Kingdom [45]	RCT	Community pharmacy	18	191 (n = 110)	EG = 73.1 (5.0) CG = 74.2 (6.3) EG = 36.4 CG = 39	Education, simplifying regime and visits/pharmacist and general practitioner	Adherence tablet count	Compliance EG = 37.6% to 47.3% and GC = 32. 0% to 14.7%; *p* = n.s.
Volume et al., 2001 Canada [46]	Cluster RCT	Community pharmacy	13	363 (n = 159)	EG = 73.89 (6.09) CG = 73.18 (6.11) EG = 36.5 CG = 30.4	Instructions, telephone call and medication review/community pharmacist	Adherence (self-reported)	Mean EG = 0.53 ± 0.77 to 0.56 ± 0.75 and CG = 0.64 ± 0.86 0.47 ± 0.69; *p* = n.s.
Wu et al., 2006 China [9]	RCT	Hospital	24	442 (n = 219)	EG = 71.2 (9.4) CG = 70.5 (11.1) EG = 48 CG = 49	Education and telephone call/clinical pharmacist	Adherence tablet count (>80% or <120%)	Became compliant = EG = 93% and CG = 82%; *p* < 0.001.

* Median (IQR), ** 0 = none, 1 = highest level; IQR = interquartile range; EG = experimental group; CG = control group; RCT = randomized clinical trial; MMAS = Morisky medication adherence scale; MARS = medication adherence report scale; MALMAS = Malaysian medication adherence scale: non-adherence (<6 score) and adherence (6–8); DPR (drug related problem) = non-compliance = two or more gaps in 20 days; n.s. = non-significant.

**Table 2:** Interventions performed and the results of the adherence to the treatment. The filled squares indicate the adherence strategies included in the study. The ✗ of the results column indicates no improvement in adherence. The ✔ of the results column indicates an improvement in adherence.

	Instructions or Counselling	Education Program or Material	Simplifying Regimen or Discharge	Reminder Systems	Monitoring Home Visits	Monitoring Visits	Monitoring Telephone	Medication Review	Medication Packages	Results
**Al-Rashed et al., 2002 [32]**										✔
**Bernsten et al., 2001 [21]**										✔
**Biswas et al., 2018 [33]**										✔
**Campins et al., 2017 [34]**										✔
**Hanlon et al., 1996 [35]**										✗
**Messerli et al., 2016 [36]**										✔
**Moral et al., 2015 [37]**										✔
**Morales et al., 2009 [38]**										✗
**Murray et al., 1993 [39]**										✔
**Muth et al., 2018 [40]**										✗
**Nazareth et al., 2001 [41]**										✗
**Odeth et al., 2019 [42]**										✔
**Sanchez-Ulayar et al., 2011 [43]**										✔
**Shim et al., 2018 [44]**										✔
**Sturgess et al., 2003 [45]**										✗
**Volume et al., 2001 [46]**										✗
**Wu et al., 2006 [9]**										✔

**Table 3:** Tools used to measure adherence to the treatment and the results of adherence. The filled squares indicate the tool used to measure adherence included in the study. The ✗ of the results column indicates no improvement in adherence. The ✔ of the results column indicates an improvement in adherence.

	Number of Medications to Be Taken/Taken	Morisky Green	Self-Report or Unspecified Questionnaire	MARS (Medication Adherence Rating Scale)	Hospitalizations or Health Care Utilization	Results
**Al-Rashed et al., 2002 [32]**						✔
**Bernsten et al., 2001 [21]**						✔
**Biswas et al., 2018 [33]**						✔
**Campins et al., 2017 [34]**						✔
**Hanlon et al., 1996 [35]**						✗
**Messerli et al., 2016 [36]**						✔
**Moral et al., 2015 [37]**						✔
**Morales et al., 2009 [38]**						✗
**Murray et al., 1993 [39]**						✔
**Muth et al., 2018 [40]**						✗
**Nazareth et al., 2001 [41]**						✗
**Odeth et al., 2019 [42]**						✔
**Sanchez-Ulayar et al., 2011 [43]**						✔
**Shim et al., 2018 [44]**						✔
**Sturgess et al., 2003 [45]**						✗
**Volume et al., 2001 [46]**						✗
**Wu et al., 2006 [9]**						✔

**Table 4:** Sources of risk of bias of randomized clinical trial.

	*Adequate Sequence Generation?*	*Allocation Concealment?*	*Blinding of Patients?*	*Blinding of Personnel?*	*Incomplete Outcome Data Addressed?*	*Free of Selective Reporting?*	*Free of Other Bias?*
Bernsten et al., 2001 [21]	-	-	-	-	-	+	+
Biswas et al., 2018 [33]	+	?	-	-	+	+	+
Campins et al., 2017 [34]	+	+	-	-	+	+	+
Hanlon et al., 1996 [35]	+	?	-	+	+	+	?
Messerli et al., 2016 [36]	+	+	-	+	+	+	+
Moral et al., 2015 [37]	-	-	-	-	+	+	+
Morales et al., 2009 [38]	?	?	-	-	+	-	+
Murray et al., 1993 [39]	?	?	-	-	+	-	?
Muth et al., 2018 [40]	+	+	-	-	+	?	+
Nazareth et al., 2001 [41]	+	?	-	-	?	+	+
Sanchez-Ulayar et al., 2011 [43]	+	+	-	?	?	+	+
Shim et al., 2018 [44]	+	?	-	+	+	+	+
Sturgess et al., 2003 [45]	?	?	-	-	+	+	+
Volume et al., 2001 [46]	-	-	-	?	-	+	?
Wu et al., 2006 [9]	+	+	-	-	-	+	+

+: low risk of bias; -: high risk of bias; ?: not clear.

**Table 5:** Sources of risk of bias of quasi-experimental studies.

	*Bias Due to Confounding*	*Bias in Selection of Participants into the Study*	*Bias of Classification of Interventions*	*Bias Due to Deviations from Intended Interventions*	*Bias Due to Missing Data*	*Bias of Measure of Outcome*	*Free of Other Bias?*
Al-Rashed et al., 2002 [32]	PN	PN	N	N	PN	N	PN
Odeh et al., 2019 [42]	Y	Y	Y	Y	PY	PY	PN

Y: Yes; PY: probably yes; PN: probably no; N: no.

**Table 6:** Results of the effect of visits and telephone call interventions (self-reported adherence (80–120%).

Study Name	Year	Events in EG	Total EG	Events in CG	Total CG	OR	CI 95% Lower	CI 95% Upper
Hanlon et al. [35]	1996	67	86	63	83	1.119	0.547	2.291
Messerli et al. [36]	2016	78	181	76	191	1.146	0.758	1.732
Nazaret et al. [41]	2001	60	131	58	135	1.122	0.691	1.820
Sanchéz-Ulayar et al. [43]	2011	29	50	8	50	7.250	2.827	18.594

EG = experimental group; CG = control group; OR = odds ratio.

**Table 7:** Results of the effects of visits and telephone calls (Morisky Green scale).

Study Name	Year	Events in EG	Total EG	Events in CG	Total CG	OR	CI 95% Lower	CI 95% Upper
Bernsten et al. [21]	2001	437	1290	449	1194	0.850	0.721	1.002
Biswas et al. [33]	2018	16	40	11	35	1.455	0.560	3.775
Moral et al. [37]	2015	33	70	33	84	1.378	0.725	2.619
Morales et al. [38]	2009	6	89	11	93	0.539	0.190	1.525
Shim et al. [44]	2018	26	51	26	79	2.120	1.030	4.365

## 4. Discussion

This systematic review summarizes the existing evidence on techniques to improve adherence in elderly polymedicated patients. Investigating the effectiveness of current adherence strategies is a complex task because several factors hamper the interpretation of the findings and, therefore, the drawing of robust conclusions. On one hand, there is a high heterogeneity among the techniques used to improve adherence, ranging from electronic tools used as reminders, pill dispensers, medication repackaging techniques and health education, to complex multidisciplinary programs that involve different health professionals and pharmacists, among others. On the other hand, another relevant aspect is the assessment of adherence itself, since, depending on the pathology or medication; studies use different units of measurement such as medication counts or adherence tests such as, for example, the “Morisky Green” or “Medication Adherence Report Scale”, drug-related problems (DRP) or even hospitalizations. Finally, there are multiple factors that influence on non-adherence, such as the patient’s age, pathology and medication regimen, the effect that the diseases and their treatments have on them, the family situation and whether they have a caregiver or live in a nursing home or alone [47]. In this review, we have attempted to provide a complete summary of the efficacy of different techniques to improve adherence, aimed at the most vulnerable patients: elderly polymedicated patients. Thus, the present study offers healthcare professionals a rigorous and practical summary of those strategies that have proven most effective in improving adherence to treatment in the over-65 population.

Our study reveals that due to the aforementioned factors, differences among the adherence strategies are very difficult to perceive, especially if complex programs with multiple interventions are used [32,33,35,36]. Thus, significant differences were only observed in three studies in which more than four interventions were applied [32,33,36]. It is necessary to consider that multidisciplinary interventions involving different professionals, such as pharmacists, nurses and primary care physicians, can be used to improve the health-related quality of care [21,32,48]. These kinds of approaches are also very useful to improve adherence as evidenced by different reviews [26,49]. In fact, the study conducted in Spain involving primary care physicians and pharmacists not only improved adherence but also the quality of life of the patients [50].

The most commonly used technique was instruction and counselling [32,33,35,36,37,38,40,41,43,46]. Another technique frequently used in the studies of our review was health education [9,21,35,36,44,45]; significant improvement in adherence to treatment has been found in all of them, except in the case of Hanlon et al. [35] and Sturgess et al. [45], two studies which showed a high risk of bias. Informing the patient about his or her pathology and the consequences of not taking the treatment is essential to observe this improvement [20]. However, replicating an educational intervention or health advice involves handling many difficulties [18], since variability depends not only on the professional, but also on the type of relationship and trust generated with the patient, as well as the type of pathology and the effects produced by the lack of adherence [25,48]. These difficulties are also observed at the economic level, as it reduces hospitalizations and consequently health care costs [51].

Another effective technique is “simplifying regime or discharge” [21,32,34,45], except in the study by Sturgess et al. [45], a study which showed a high risk of bias. However, all studies using a medication review revealed unfavorable results [40,41,46]. Interestingly, study conducted by Lipton et al. [52] which claimed that drug reviews reduced the percentage of patients with DRP with respect to the control group (83% of intervention patients and 92% of control patients). Likewise, Willeboordse et al. [53] showed an improvement in DRP, even though polymedicated patients were not included, suggesting that this strategy could be more effective in patients with few prescribed drugs.

Pharmacist accompaniment improves confidence and knowledge and offers long-term support and security to patients [18,48]. This may be one of the reasons why no differences were found in the Morales et al. study [38], in which pill dispensers were used, but no such accompaniment was applied. A slight improvement in adherence was also observed in the study by Murray et al. [39] where there was also no pharmacist collaboration.

The fundamental bias identified in all the studies was the lack of patient and investigator blinding. In the case of patients, blinding would be especially important due to the Hawthorne effect [54] which consists of the tendency to improve compliance if the patients feel that they are being observed. One way to avoid the Hawthorne effect is blinding the studies to the patient by providing them with an information sheet or brief advice. Another of the biases identified was the blinding of the allocation. The latter is very important to prevent and thereby an explanation about the actions taken to carry out this blinding should be included in the studies. In general, these studies show a low methodological quality with a high risk of bias.

There are currently several reviews that have measured adherence to medications [18,20,24,25,29]. However, most of the studies have focused on patients taking a single drug or group of drugs, so the majority of them evaluated the improvement of adherence to a single drug or group of drugs [55]. This fact may limit the applicability to the general elderly population. In other cases, the revisions are also quite old (more than 15 years old) [18,20,24,25]. Furthermore, in most of the previous studies the age was not restricted to people over 65 years old and their inclusion criteria were broader. That is, the mean age was 65 years old or more, which means that there were several patients under the age of 65 [27]. Therefore, the applicability of the evaluated techniques to improve adherence may vary substantially, as age influences adherence and compliance with medication [56]. In addition, our target population is more likely to have comorbidity, a higher number of medications and cognitive difficulties [57,58].

In the evaluated studies, we have found certain limitations in the follow-up of the techniques, as it is important to be able to evaluate them over a long period to know their long-term effect. Thus, although an effect could be observed after intervention, this effect may disappear as time goes by [53]. On the other hand, no data were collected on the existence of cohabitants, who may help the elderly to take their medication, motivate the patient, participate in the counting of the medication and, in short, have a positive influence on therapeutic adherence [59].

The data obtained from the meta-analysis indicated that adherence to treatment improves with visits or telephone call from a health professional when measured with the Morisky scale, as previously shown by other authors who studied adherence to asthma treatment [60,61]. However, when using non-validated and self-reported questionnaires, no differences were found. Validated scales are a valuable tool that allows the systematic collection and analysis of a large amount of information in an easy and inexpensive way. Compared to interviews, they have the advantage of eliminating bias introduced by the influence of the interviewer, as well as quantifying and universalizing information to facilitate the comparability of data [62].

In short, the elderly population has specific characteristics, including polymedication. Most of the studies developed to measure adherence and the efficacy of different interventions in improving adherence are aimed at specific pathologies or treatments, which does not allow us to extrapolate the data to the polymedicated population. Furthermore, the studies aimed at resolving this issue have a large number of variables, and although the efficacy of the same intervention is measured in different studies, the measure of adherence is different in each of the studies. We recommend the use of validated scales instead of self-reported scales; as can be seen in the meta-analysis, this gives us the opportunity to better observe the differences between groups. This affects the possibility of obtaining clear conclusions regarding the efficacy of interventions to improve adherence to treatment. Furthermore, in many cases the studies were short in duration, which does not allow long-term results to be observed.

## 5. Conclusions

In conclusion, in all studies that combined at least instructions or advice to the patient on the use of pharmacological treatment with follow-up, through visits or calls, and which measured adherence directly or with validated scales, an improvement in adherence to treatment was observed. However, given the variability of the strategies used in the different studies analyzed, and the fact that in most cases they were used in combination, it was impossible to determine the efficacy of any of the strategies analyzed. On the other hand, the measurement tools used to improve adherence is variable, which makes it difficult to compare the results of different studies. On the other hand, the meta-analysis reveals that monitoring by visits or telephone call is effective on the Morisky Green scale. Innovative strategies to improve medication adherence in the elderly and reliable measures of adherence are needed, as well as studies of higher methodological quality. Until more evidence of single-intervention strategies is available, we cannot conclude the efficacy of the strategies analyzed.

## Data Availability

Not applicable.

## Supplementary Materials

The following supporting information can be downloaded at: https://www.mdpi.com/article/10.3390/pharmacy12010035/s1, Table S1. Search strategies.
