# Supplementary material for: Strategies to Improve Therapeutic Adherence in Polymedicated Patients over 65 Years: A Systematic Review and Meta-Analysis
Source: Pharmacy (Basel). 2024 Feb 17;12(1):35. doi: 10.3390/pharmacy12010035 (PMC10892390; doi:10.3390/pharmacy12010035)
Supplement: Supplementary file 1 [file pharmacy-12-00035-s001.zip › pharmacy-2762852-suplementary.pdf]

Supplementary Materials:

Table S1. Search strategies.

Electronic databases	Search
**PubMed**	(therapeutics OR treatments OR therapy OR treatment OR medication OR drug) AND (adherance OR adhere OR adhered OR adherent OR adherers OR adhering OR compliances OR patient compliance) AND (interventions OR interventive methods OR strategy OR strategies) AND polypharmacy AND (older OR elder OR elderly OR elderlies)<br>Limit: Aged
**Cochrane Central Register of systematic reviews.**	TS, TI: adherence OR compliance AND TS, TI: intervention OR strategy AND TS, TI: polypharmacy AND TS TI older OR aged OR elderly
**Web of science**	(**treatment** OR **medication** OR **drug** (Topic) and **adherence OR compliance** (Topic) and **intervention OR strategy** (Topic) and **polypharmacy** (Topic) and **older OR aged OR elderly** (Topic)

TS: Key word/ TI:Title and abstract
